# Supplementary material for: Reconciling Mining with the Conservation of Cave Biodiversity: A Quantitative Baseline to Help Establish Conservation Priorities
Source: PLoS One. 2016 Dec 20;11(12):e0168348. doi: 10.1371/journal.pone.0168348 (PMC5173368; doi:10.1371/journal.pone.0168348)
Supplement: S1 Dataset — (ZIP) [file pone.0168348.s002.zip › Taxa/Serra Norte/SN_2013/N4E/Lista N4E-73.pdf]

CAVIDADE N4E-0073

Classe	Ordem	Fam/Outros	Gên/Outros	Espécie	S-U	U-S	Zon.
Annelida	Hirudinea			sp.1		2	P
Annelida	Oligochaeta			jovem	1	7	E/P
Arachnida	Acari	(Oribatida)		sp.3		X	E
Arachnida	Acari	(Parasitengonina)		sp.10		X	E
Arachnida	Acari	(Parasitengonina)		sp.2		X	E
Arachnida	Acari	Anystidae	*Erythracarus*	sp.1		X	P
Arachnida	Acari	Asternoseiidae		sp.84	X		
Arachnida	Acari	Erythraeidae		sp.64	X		P
Arachnida	Acari	Eupodidae		sp.1	X		E
Arachnida	Acari	Gamasida		jovem	X		
Arachnida	Acari	Ixodidae	*Amblyomma*	sp.1		X	E
Arachnida	Acari	Labidostommatidae		sp.1	X		E
Arachnida	Acari	Laelapidae		sp.1		X	P
Arachnida	Acari	Laelapidae		sp.56	X		E
Arachnida	Acari	Mesostigmata		jovem	X		E
Arachnida	Acari	Prostigmata		sp.49	X		P
Arachnida	Acari	Trombiculidae		sp.39		X	P
Arachnida	Acari	Veigaiidae		sp.1		X	E/P
Arachnida	Amblypygi	Charinidae	*Charinus*	sp.1	3	2	P
Arachnida	Amblypygi	Phrynidae	*Heterophrynus*	*longicornis*	4	1	E/P
Arachnida	Araneae	Araneidae		jovem	X		E/P
Arachnida	Araneae	Caponiidae		sp.1	X	X	P
Arachnida	Araneae	Corinnidae	*Creugas*	sp.1		1	P
Arachnida	Araneae	Ctenidae		jovem	1	1	E/P
Arachnida	Araneae	Drymusidae	*Drymusa*	*spelunca*	X	X	E
Arachnida	Araneae	Hahniidae		sp.1	X	X	E
Arachnida	Araneae	Idiopidae		jovem	1		P
Arachnida	Araneae	Linyphiidae		jovem		X	P
Arachnida	Araneae	Mysmenidae	*Microdipoena*	sp.1	X		E/P
Arachnida	Araneae	Nesticidae	*Nesticus*	sp.2	X	X	P
Arachnida	Araneae	Ochyroceratidae	*Ochyrocera*	sp.2	X		P
Arachnida	Araneae	Ochyroceratidae	*Speocera*	sp.1	X	X	E/P
Arachnida	Araneae	Oonopidae	*Neoxyphinus*	sp.2		X	E
Arachnida	Araneae	Oonopidae	*Neoxyphinus*	sp.3	X		P
Arachnida	Araneae	Pholcidae	*Leptopholcus*	sp.1	X		E
Arachnida	Araneae	Pholcidae	*Mesabolivar*	*aurantiacus*		X	P
Arachnida	Araneae	Pholcidae	*Mesabolivar*	sp.1	X	X	E/P
Arachnida	Araneae	Scytodidae	*Scytodes*	jovem		1	E
Arachnida	Araneae	Tetragnathidae		jovem	X		E
Arachnida	Araneae	Theraphosidae		jovem	1		E/P
Arachnida	Araneae	Theridiidae	*Theridion*	sp.1	X	X	E
Arachnida	Araneae	Theridiosomatidae	*Plato*	sp.1	X	X	E/P
Arachnida	Araneae	Uloboridae	*Uloborus*	sp.3	X		E
Arachnida	Opiliones	Cosmetidae	*Roquettea*	*singularis*	1		E/P
Arachnida	Opiliones	Sclerosomatidae	*Prionostema*	sp.1	7	1	E/P
Arachnida	Palpigradi	Eukoeneniidae	*Eukoenenia*	sp.1	X	X	E/P
Arachnida	Pseudoscorpiones	Bochicidae		sp.1	X		P
Arachnida	Pseudoscorpiones	Chthoniidae		jovem		X	E/P
Arachnida	Schizomida	Hubbardiidae		jovem		X	P

Chilopoda	Geophilomorpha	Geophilidae		sp.1	1	1	A
Chilopoda	Scolopendromorpha	Cryptopidae	*Cryptops*	jovem	4	3	E/P
Chilopoda	Scolopendromorpha	Scolopocryptopidae	*Tidops*	sp.1		1	P
Chilopoda	Scutigeromorpha	Pselliodidae	*Sphendononema*	*guildingii*	2		E/P
Diplopoda	Polydesmida	Chelodesmidae		sp.3	4	1	P
Diplopoda	Polydesmida	Cyrtodesmidae		sp.1	X	X	P
Diplopoda	Polydesmida	Fuhrmannodesmidae		sp.1	X	X	E/P
Diplopoda	Polydesmida	Paradoxosomatidae		sp.1	1	1	E
Diplopoda	Polydesmida	Pyrgodesmidae		sp.1	X	X	P/A
Diplopoda	Spirostreptida	Pseudonannolenidae	*Pseudonannolene*	jovem		1	E
Entognatha	Collembola	Entomobryidae		sp.1	X		E/P
Entognatha	Collembola	Entomobryidae		sp.6	X		P
Entognatha	Collembola	Entomobryidae		sp.8	X		P
Entognatha	Collembola	Isotomidae		sp.1	X		P
Entognatha	Collembola	Paronellidae		sp.1	X	X	E/P/A
Entognatha	Collembola	Paronellidae		sp.4	X	X	E/P/A
Entognatha	Collembola	Paronellidae		sp.6		X	E/P
Entognatha	Collembola	Sminthuroidea		sp.2	X	X	P
Entognatha	Collembola	Sminthuroidea		sp.3		X	P
Entognatha	Diplura	Campodeidae		sp.1	X	X	E/P
Entognatha	Diplura	Japygidae		sp.1	X	X	E
Insecta	Blattodea	Blattellidae		jovem	2	1	E/P
Insecta	Coleoptera	Carabidae		sp.3		X	P
Insecta	Coleoptera	Curculionidae	Scolytinae	sp.3	X		P
Insecta	Coleoptera	Dytiscidae		sp.12A		X	P
Insecta	Coleoptera	Dytiscidae		sp.7		X	P
Insecta	Coleoptera	Scydmaenidae		sp.1		X	P
Insecta	Coleoptera	Staphylinidae	Pselaphinae	sp.28		X	P
Insecta	Coleoptera	Staphylinidae	Pselaphinae	sp.34	X		E
Insecta	Coleoptera	Staphylinidae	Pselaphinae	sp.37	X		P
Insecta	Diptera	Cecidomyiidae	Cecidomyiinae	sp.		X	P
Insecta	Diptera	Chironomidae		sp.	X		P
Insecta	Diptera	Culicidae	*Culex*	sp.	X	X	P
Insecta	Diptera	Phoridae		sp.		X	E
Insecta	Diptera	Psychodidae	Phlebotominae	sp.	X	X	P
Insecta	Diptera	Tipulidae	Tipulinae	sp.	X	X	E/P
Insecta	Heteroptera	Cydnidae		jovem	X		A
Insecta	Heteroptera	Reduviidae	Emesinae	sp.	1		E
Insecta	Heteroptera	Reduviidae	Reduviinae	sp.	1		E/P
Insecta	Heteroptera	Reduviidae	Triatominae	sp.	1	1	P
Insecta	Homoptera	Cixiidae		jovem	X	X	E/P
Insecta	Hymenoptera	Formicidae	*Camponotus*	sp.1		X	P
Insecta	Hymenoptera	Formicidae	*Camponotus*	sp.2		X	P
Insecta	Hymenoptera	Formicidae	*Eurhopalothrix*	sp.1	X	X	E
Insecta	Hymenoptera	Formicidae	*Odontomachus*	sp.1		X	E
Insecta	Hymenoptera	Formicidae	*Pachycondyla*	sp.2	X	X	E/P
Insecta	Hymenoptera	Formicidae	*Paratrechina*	sp.1	X		P/A
Insecta	Hymenoptera	Formicidae	*Paratrechina*	sp.2	X		P
Insecta	Hymenoptera	Formicidae	*Solenopsis*	sp.1		X	E
Insecta	Isoptera	Rhinotermitidae	*Heterotermes*	sp.	X		P
Insecta	Isoptera	Termitidae	*Nasutitermes*	sp.	X	X	E/P
Insecta	Lepidoptera	Noctuoidea		sp.1	1		P

Insecta	Lepidoptera	Noctuoidea		sp.7		1	E
Insecta	Lepidoptera	Tineoidea		sp.1		X	E
Insecta	Orthoptera	Phalangopsidae	*Paraclodes*	sp.	6		E/P
Insecta	Orthoptera	Phalangopsidae	*Phalangopsis*	sp.	56	20	P/A
Insecta	Psocoptera	Epipsocidae	*Epipsocus*	sp.1	X	X	P
Insecta	Thysanura	Ateluridae		jovem	X		A
Insecta	Thysanura	Nicoletiidae		sp.1	X	X	P
Gastropoda	Pulmonata	Bulimulidae	*Naesiotus*	sp.	X	X	P/A
Gastropoda	Pulmonata	Subulinidae		sp.5		X	P
Gastropoda	Pulmonata	Systrophiidae	*Happia*	sp.	X	X	E/P
Malacostraca	Isopoda	Armadillidae		sp.1	X	X	E/P
Malacostraca	Isopoda	Dubioniscidae		sp.1		X	E
Malacostraca	Isopoda	Philosciidae		sp.1	X		E/P
Malacostraca	Isopoda	Philosciidae		sp.2		X	E
Malacostraca	Isopoda	Platyarthridae		sp.1		X	E/P
Malacostraca	Isopoda	Scleropactidae	*Circoniscus*	*buckupi*	X	X	E/P/A
Malacostraca	Isopoda	Scleropactidae		sp.1		X	P
Symphyla		Scutigerellidae		sp.	X	X	E/P
Turbellaria	Tricladida			sp.1		1	P
Amphibia	Anura	Dendrobatidae	*Epipedobates*	sp.	1		E
Amphibia	Anura	Strabomantidae	*Pristimantis*	cf. *fenestratus*	9	1	E/P/A
Mammalia	Chiroptera	Emballonuridae	*Peropteryx*	*kappleri*	10	2	P/A
Mammalia	Chiroptera	Furipteridae	*Furipterus*	*horrens*	6	3	P/A
Mammalia	Rodentia	Cricetidae	*Rhipidomys*	sp.	2		P/A
Reptilia	Squamata	Gekkonidae	*Thecadactylus*	sp.		2	E
